# A Hipotensão Ortostática Infrequente no Brasil: Estamos Subestimando o Problema?

**DOI:** 10.36660/abc.20200352

**Published:** 2020-06-29

**Authors:** Humberto Graner Moreira

**Affiliations:** 1 Liga de Hipertensão Arterial Faculdade de Medicina Universidade Federal de Goiás GoiâniaGO Brasil Liga de Hipertensão Arterial (LHA) - Faculdade de Medicina da Universidade Federal de Goiás,Goiânia, GO – Brasil

**Keywords:** Hipotensão Ortostática/complicações, Epidemiologia, Doenças Cardiovasculares, Acidente Vascular Cerebral, Infarto do Miocárdio, Hipertensão, Saúde do Adulto

A homeostase da pressão arterial (PA) depende de mecanismos fisiológicos complexos que envolvem interações contínuas dos sistemas cardiovascular, renal, neural e endócrino. Esses mecanismos também devem garantir a manutenção do débito cardíaco adequado, mesmo em situações de rápidas variações circulatórias. Uma dessas situações está relacionada às mudanças dinâmicas da postura, de deitado para em pé, quando a rápida redução do retorno venoso pode afetar a pré-carga, o volume sistólico e a pressão arterial média. A hipotensão ortostática (HO) é uma manifestação de disfunção autonômica e ocorre quando os mecanismos adaptativos cardiovasculares falham em compensar essas alterações ao assumir a posição em pé.^1^

O diagnóstico de HO requer a demonstração de diminuição significativa e persistente da PA durante a ortostase, seja pelo teste de se levantar ativamente à beira leito ou pelo teste de inclinação (tilt-test). Diretrizes nacionais e internacionais endossam a definição de HO como uma queda ≥20 mmHg na pressão arterial sistólica (PAS) ou uma queda ≥10 mmHg na pressão arterial diastólica (PAD) dentro de 3 minutos após a posição ortostática, independentemente da presença de sintomas.^2 - 4^ Essa definição foi estabelecida pela primeira vez por um consenso em 1996 e foi baseada em vários pequenos estudos fisiológicos, além de considerações pragmáticas.^5^ Com essa definição, evidências crescentes têm demonstrado que a HO prediz mortalidade por todas as causas^6 , 7^ e incidência de doenças cardiovasculares,^7 , 8^ sendo ainda mais relevante do que o descenso noturno reverso, observada na monitorização ambulatorial da PA, para predizer eventos cardiovasculares.^9^ Uma meta-análise recente envolvendo 121.913 indivíduos e um acompanhamento médio de 6 anos relatou que a HO estava associada a riscos 50, 41 e 64% maiores de morte por todas as causas, doença arterial coronariana e acidente vascular cerebral, respectivamente.^8^

Para determinar a prevalência de HO em uma população brasileira, Velten e colegas apresentam nesta edição dos Arquivos uma análise detalhada do comportamento da pressão arterial após manobras posturais em 14.833 indivíduos do estudo ELSA-Brasil.^10^ A coorte ELSA-Brasil incluiu 15.105 funcionários públicos de 35 a 74 anos de 5 universidades e 1 instituto de pesquisa localizados em diferentes regiões do Brasil. O estudo foi realizado de 2008 a 2010 e desenvolvido para abordar a incidência de doenças cardiovasculares e os principais fatores de risco associados entre funcionários funcionários ativos ou aposentados dessas instituições.^11^ A prevalência relatada de HO nessa população foi de 2,0% e aumentou com a idade, chegando a 3,3% em indivíduos entre 65 e 74 anos. Entre aqueles com triagem positiva para HO, a presença de sintomas foi observada em 19,7%, contra apenas 1,4% entre aqueles sem HO. Os sintomas foram relatados em até 43% dos indivíduos que apresentaram queda concomitante na PAS e PAD.

Para além de um inquérito epidemiológico, em um país onde muitos desses dados são escassos ou ausentes, este estudo levanta algumas questões que merecem ser abordadas. Primeiro, a prevalência de HO nesta coorte foi baixa. Infelizmente, não existem outros estudos na literatura que tenham investigado a prevalência de HO no Brasil, e esse também é outro mérito dos autores. Pesquisas epidemiológicas internacionais demonstram que a prevalência de HO varia de 5 a 20%, mas pode chegar a 30% em indivíduos com mais de 70 anos de idade.^1 , 12^ Neste trabalho de Velten et al.,^10^ mesmo em indivíduos com mais de 64 anos, a prevalência ainda era muito menor do que os relatos anteriores. As razões para essa discrepância não foram claras. Uma parcela significativa dos idosos acima de 74 anos foi excluída e poderia aumentar esse número, mas as características basais do estudo ELSA-Brasil ainda apontam para uma população com alta frequência de fatores de risco: 63,1% tinham excesso de peso, 61,5% apresentavam colesterol alto, 35,8% apresentavam PA elevada, e 20,3% tinham tolerância à glicose comprometida.^11^ Se a baixa prevalência apenas reflete uma população específica, retomaremos esse tópico mais adiante neste artigo.

Segundo, como parte da avaliação do protocolo, a manobra de mudança postural incluiu medidas da PA aos 2, 3 e 5 minutos após o repouso. Os autores apontam que a prevalência de HO pode mais que dobrar, para até 4,3%, quando se considera a redução da PA em pelo menos uma das três medidas. No entanto, ao comparar apenas as medidas de 3 e 5 minutos, a prevalência de HO aumenta de 2,0 para 2,6%. Embora esses indivíduos tendam a ser mais sintomáticos aos 5 minutos, o aumento da sensibilidade para a triagem de HO é pequeno, e certamente não justifica estender as medidas além de 3 minutos durante uma avaliação no consultório.

Mas talvez um dos aspectos mais interessantes deste trabalho tenha sido o cálculo dos escores Z para variações da PA, observando valores inferiores aos estabelecidos pelas diretrizes para um subconjunto específico da população. A distribuição da variação da PA resultou em escores Z -2 de -14,09 mmHg para PAS e -5,39 mmHg para PAD na subamostra de pacientes sem hipertensão, diabetes, história de insuficiência cardíaca, doença arterial coronariana, infarto do miocárdio prévio ou acidente vascular cerebral. Isso significa que, nesta coorte de adultos brasileiros, os atuais limiares nacionais e internacionais podem subestimar a presença de HO. Essa diferença pode, inclusive, explicar a menor prevalência nessa coorte brasileira. Como existem reflexos autonômicos envolvidos na resposta fisiológica da PA em pé,^1^ é razoável admitir que poderíamos ter variações diferentes para diferentes populações. Em outras palavras, um número não pode servir para todos. O estudo de Velten et al.^10^ fornece dados para uma discussão mais ampla sobre esse assunto. Obviamente, mais dados serão necessários em populações diversas, uma vez que o estudo ELSA-Brasil avaliou apenas uma amostra específica de funcionários de seis instituições brasileiras.

Independentemente se entraremos em discussões sobre os limiares de HO no país — se uma queda de 20 ou 14 mmHg na PAS —, isso não muda o fato de que o problema pode continuar sendo negligenciado na prática clínica. Existe uma recomendação formal para medir a PA no 1º e 3º minutos após ficar em pé, partindo de uma posição sentada, em todos os pacientes na primeira avaliação no consultório para identificar a HO.^2 - 4^ As medidas da PA em repouso e em pé também devem ser consideradas em visitas subsequentes em pacientes idosos, diabéticos e pessoas com outras condições em que a HO pode ocorrer com frequência. No entanto, mesmo sabendo das suas possíveis implicações na incidência de eventos cardiovasculares, a HO é frequentemente subdiagnosticada e pode ser um problema negligenciado na prática clínica.

Aproximadamente dois terços dos pacientes com HO não serão detectados se as medidas sequenciais da PA na posição vertical não forem realizadas na prática.^13^ Mesmo em um estudo clínico desenvolvido para avaliar a eficácia da monitorização ambulatorial da PA na detecção da HO, apenas 76% dos 505 pacientes foram triados durante visitas regulares ao consultório.^14^ A falta de tempo durante as consultas pode ser um dos principais fatores. Além disso, agora pode-se argumentar que a prevalência de HO em indivíduos de meia-idade é realmente baixa, questionando se devemos realizar uma triagem sistemática conforme recomendado. No entanto, não há dúvidas sobre as implicações prognósticas da HO, principalmente em idosos. Talvez essa discussão sobre hipotensão postural mereça a devida atenção para melhorar nossa sensibilidade, identificando quem realmente precisa ser avaliado e quais seriam as variações esperadas da PA para cada grupo de indivíduos.
