# Supplementary material for: A Hipotensão Ortostática Infrequente no Brasil: Estamos Subestimando o Problema?
Source: Arq Bras Cardiol. 2020 Jun 29;114(6):1049–50. [Article in Portuguese] doi: 10.36660/abc.20200352 (PMC8416131; doi:10.36660/abc.20200352)
Supplement: Translation

# Translation: The Uncommon Orthostatic Hypotension in Brazil: Are We Underestimating the Problem?

**Authors:** Humberto Graner Moreira

**Affiliations:** 1 Brazil Liga de Hipertensão Arterial (LHA) - Faculdade de Medicina da Universidade Federal de Goiás, Goiânia, GO – Brazil

**Keywords:** Hypotension, Orthostatic/complications, Epidemiology, Cardiovascular Diseases, Stroke, Myocardial Infarction, Hypertension, Adult, Health

Blood pressure (BP) homeostasis depends on complex physiological mechanisms that involve continuous interactions of the cardiovascular, renal, neural, and endocrine systems. These mechanisms must also guarantee the maintenance of adequate cardiac output, even in situations of rapid circulatory variations. One of these situations is related to the dynamic posture changes, from lying to standing, when the rapid reduction in venous return can affect preload, stroke volume, and mean BP. Orthostatic hypotension (OH) is a manifestation of autonomic dysfunction and occurs when cardiovascular adaptive mechanisms fail to compensate for those changes when assuming the standing position.^1^

Diagnosing OH requires the demonstration of significant persistent BP decrease during orthostasis, either by the bedside active-standing test or a tilting test. National and international guidelines have endorsed the definition of OH as a ≥20 mmHg drop in systolic blood pressure (SBP) or a ≥10 mmHg drop in diastolic blood pressure (DBP) within 3 minutes after standing, regardless of the presence of symptoms.^2 - 4^ This definition was first established by a consensus in 1996 and was based on several small physiology studies as well as on pragmatic considerations.^5^ Upon this definition, growing evidence has been shown that OH predicts all-cause mortality^6 , 7^ and incidence of cardiovascular disease,^7 , 8^ being even more relevant than the ambulatory BP monitoring-derived nighttime reverse dipping for predicting cardiovascular events^9^ . A recent meta-analysis involving 121,913 individuals and a median follow-up of 6 years reported that OH was associated with a 50, 41, and 64% greater risks of all-cause death, coronary heart disease, and stroke, respectively.^8^

In order to determine the prevalence of OH in a Brazilian population, Velten and colleagues present in this issue of the Archives a detailed analysis of the blood pressure behavior following postural maneuvers in 14,833 individuals from the ELSA-Brasil study.^10^ The ELSA-Brasil cohort included 15,105 civil servants aged 35 to 74 years old from 5 universities and 1 research institute located in different regions of Brazil. The study was carried out from 2008 through 2010 and was designed to address the incidence of cardiovascular diseases and major associated risk factors among active or retired employees from those institutions.^11^ The reported prevalence of OH in this population was 2.0%, and increasing with age, reaching up to 3.3% in individuals between 65 and 74 years old. Among those with positive screening for OH, the presence of symptoms was noted in 19.7 vs. only 1.4% among those without OH. Symptoms were reported in up to 43% of individuals who had a concomitant fall in SBP and DBP.

For beyond an epidemiological study, in a country where much of these data are scarce or absent, this study raises some issues that deserve to be addressed. First, the prevalence of OH in this cohort was low. Unfortunately, there are no other studies in the literature that have investigated the prevalence of OH in Brazil, and this is also another merit of the authors. International epidemiologic surveys have found that the prevalence of OH varies from 5 to 20 percent but can reach up to 30% in individuals over 70 years of age.^1 , 12^ The prevalence was still much lower in this work by Velten et al.^10^ than in previous reports, even in individuals aged over 64 years. The reasons for this discrepancy were not clear. A significant portion of the elderly beyond the age of 74 were excluded and could raise this number, but the baseline characteristics of the ELSA-Brasil study still pointed to a population with a high frequency of risk factors: 63.1% were overweight, 61.5% had high cholesterol, 35.8% presented with high blood pressure, and 20.3% had impaired glucose tolerance.^11^ If the low prevalence could only reflect a specific population, this topic will be resumed later in this paper.

Second, as part of the protocol assessment, the postural change maneuver included BP measurements at 2, 3, and 5 minutes after standing. The authors point out that the prevalence of OH could more than double to up to 4.3% when considering the reduction in BP in at least one of the three measurements. However, when comparing only the 3- and 5-minute measurements, the prevalence of OH rises from 2.0 to 2.6%. Even though these individuals tend to be more symptomatic at 5 minutes, the increase in sensitivity for screening is small, and certainly does not justify extending the measurements beyond 3 minutes during an office evaluation.

But perhaps one of the most interesting aspects of this work was the calculation of Z-scores for BP variations, observing values lower than those established by guidelines for a specific subset of the population. The distribution of BP variation resulted in -2 Z-scores of -14.09 mmHg for SBP and -5.39 mmHg for DBP in the subsample of patients without hypertension, diabetes, history of heart failure, coronary heart disease, previous myocardial infarction, or stroke. This means that, in this cohort of Brazilian adults, the current national and international thresholds may underestimate the presence of OH. This difference could even explain its lower prevalence in this Brazilian cohort. Since there are autonomic reflexes involved in the physiologic blood pressure response upon standing,^1^ it is reasonable to admit that we could have different variations for different populations. In other words, one number could not fit for all. The study by Velten et al.^10^ provides data for a broader discussion regarding this issue. Obviously, more data will be needed in diverse populations, since the ELSA-Brasil study evaluated only a specific sample of employees from six Brazilian institutions.

Regardless of whether to engage into discussions about the thresholds for OH in the country — if a drop of 20 or 14 mmHg in SBP —, this does not change the fact that the problem could continue to be neglected in clinical practice. There is a formal recommendation to measure BP 1 minute and 3 minutes after standing from a seated position in all patients at the first office evaluation to address OH.^2 - 4^ Lying and standing BP measurements should also be considered in subsequent visits in elderly, diabetic patients, and people with other conditions in which orthostatic hypotension may frequently occur. However, even knowing the possible implications for the incidence of cardiovascular events, OH is often misdiagnosed and may be an overlooked issue in clinical practice.

Approximately two-thirds of patients with OH could not be detected if sequential BP measurements at upright position are not performed in common practice.^13^ Even in a clinical study designed to evaluate the effectiveness of ambulatory BP monitor in detecting OH, only 76% of the 505 patients were screened during regular office visits.^14^ Lack of time during consultations could be one of the main factors. In addition, it can now be argued that the OH prevalence in middle-aged individuals is indeed low, questioning whether we should perform systematic screening as recommended. Nevertheless, there are no doubts about the prognostic implications of OH, especially in the very elderly. Perhaps this discussion about postural hypotension deserves due attention in order to improve our sensitivity by identifying who really needs to be evaluated and what would be the expected BP variations for each group of individuals.
